# Evaluation of a Sentinel Hypertension Surveillance System in Mojo, East Shewa Zone, Oromia, Ethiopia: Concurrently Embedded Mixed Design Study

**DOI:** 10.2196/72909

**Published:** 2025-06-03

**Authors:** Abiyie Demelash Gashe, Yeshiwas Ayale Ferede, Dawit Zenebe Weldemichael, Aman Yesuf Endries

**Affiliations:** 1 Ethiopian Field Epidemiology Training Program, Department of Epidemiology, School of Public Health, St. Paul's Hospital Millennium Medical College, Addis Ababa, 1000, Ethiopia, 251945142918; 2 Department of Reproductive Health, Teda Health Science College, Gondar, Ethiopia; 3 Department of Epidemiology, College of Health Science, Mekelle University, Mek'ele, Ethiopia; 4 Department of Epidemiology, School of Public Health, St. Paul's Hospital Millennium Medical College, Addis Ababa, Ethiopia

**Keywords:** hypertension, sentinel surveillance, public health surveillance, noncommunicable disease surveillance, Ethiopia

## Abstract

**Background:**

In response to the increasing incidence and prevalence of hypertension, Ethiopia has been piloting hypertension control at the primary health care level in selected sentinel sites. However, no evaluation has been conducted and its success and failures have not been ascertained.

**Objective:**

This study aimed to evaluate on whether sentinel hypertension surveillance system in Mojo City were operating efficiently and effectively.

**Methods:**

A concurrently embedded mixed design (quantitative or qualitative) study was conducted in 2 sentinel health centers in Mojo city, Oromia region of Ethiopia. The usefulness and 9 system attributes were assessed via key informant interviews, observations, and record reviews. The qualitative data were analyzed manually via thematic analysis, whereas quantitative data were analyzed via SPSS Software version 25.0 (IBM Corp).

**Results:**

The study invited 14 key informants, and all were willing to participate in the interview. The completeness and timeliness of reports were 98% and 100%, respectively. The sensitivity, positive predictive value, and representativeness were 45.3%, 92.6%, and 22%, respectively. Nearly three-fourths (10/14, 71%) of key informants perceived the system as flexible, while half thought it as unstable due to factors such as inadequate training and lack of supportive supervision and feedback system. Health facilities did not conduct routine data analysis and interpretation, nor did they use for action.

**Conclusions:**

The surveillance system in Mojo city was simple, flexible, acceptable, and predictive but less sensitive, unrepresentative, and unstable. There is a need for implementing routine data analysis and use for action, adequate training, and feedback system for optimizing the system’s performance and to ensure its sustainability.

## Introduction

Hypertension is the leading risk factor for cardiovascular diseases [1,2]. Approximately 1.28 billion adults aged 30 to 79 years have hypertension worldwide, with low- and middle-income countries bearing two-thirds of the global burden. Alarmingly, 46% of adults with hypertension are unaware of their condition while only 42% received appropriate diagnosis and treatment. Furthermore, 1 in 5 individuals with hypertension have successfully controlled their blood pressure (BP) [3]

While global trends in hypertension have shown significant increases each year, the rise is disproportionately seen in low- and middle-income countries especially in the African region [4,5]. By the end of 2021, the World Health Organization (WHO) of Africa exhibited the highest prevalence of hypertension (27%) compared with other regions [3]. In sub-Saharan Africa, the prevalence has been reported as high as 48% [5].

Hypertension is among one of the rising noncommunicable diseases (NCDs) [6-8] due to factors such as rapid urbanization, aging population as result of improved life expectancy, globalization of marketing and trade, and exposure to the social determinants of health [9,10]. The WHO estimated that the prevalence of hypertension in Ethiopia exceeds 20% and is alarmingly increasing over time [11]. Data from meta-analyses indicate that the prevalence of hypertension ranges from 9.3% to 30.3% in population-based studies, 7% to 37% in institution-based studies, and 13.2%% to 18.8% in hospital-based studies [12]. A substantial gap also exists in hypertension control in the country, with several hospital-based studies reporting BP control rates ranging from 26.4% to 52.5% [13-16].

Due to the rising incidence and prevalence of NCDs including hypertension, strengthening of the NCD surveillance system has become increasingly important. The WHO has emphasized the need for improved NCD surveillance systems to effectively tackle NCDs [17]. Consequently, Ethiopia has made a significant shift in its approach to public health surveillance. For over 3 decades, the country implemented surveillance of epidemic-prone communicable diseases under public health emergency management system [18]. However, country adopted its first NCD-inclusive public health emergency management guideline that included hypertension and diabetes mellitus by 2021 [19].

In addition, to enhance hypertension prevention and control, the Federal Ministry of Health, in collaboration with the WHO and Resolve to Save Lives, initiated a hypertension control project at the primary health care level including 74 health facilities and 200 health posts since 2019 [20]. These facilities have been used as hypertension sentinel sites to facilitate early detection, timely response, and data-driven decision making.

The target population for this surveillance system comprises adults aged 30 years and older. The major milestones of this initiative include screening 30% of the eligible population and achieving a 50% BP control. However, no evaluation has been conducted at either national or subnational levels. Hence, evaluating this program is crucial to ascertain its success and failures, share lessons learnt, guide evidence-based decision making, inform clinical practice, and improve overall hypertension control. Therefore, this study aimed to evaluate on whether the sentinel hypertension surveillance systems in 2 health centers in Mojo City, Oromia region of Ethiopia are operating effectively and efficiently.

## Methods

### Study Area

Mojo City is located in the East Shewa zone, Oromia region of Ethiopia at 74 km distance from Addis Ababa, the capital city of Ethiopia. Based on Ethiopia’s 2007 census data, the estimated population of Mojo City was 58,941, of which 51% (30,060) were females. Adults aged 30 years and older constitute 29% (17,093/58,941) of the total population. Mojo City has 1 primary hospital, 2 health centers, and 2 health posts. Mojo City is strategically located along the Ethio-Djibouti railway and was identified by the Ministry as one of the high NCD burden areas in the country.

### Study Design

This study used a concurrently embedded a mixed methods approach that combines both quantitative and qualitative data collection strategies at 2 sentinel sites in Mojo City, such as Mojo and Haachaltu Gudina Health Centers, and their 2 administrative institutions, such as the Mojo City health administration office and Oromia regional health Bearau. The evaluation included the performances of these sites from September 1, 2021 to July 30, 2022.

### Sampling Procedures

We purposively included the only available 2 sentinel sites in Mojo City, that is, Mojo and Haachaltu Gudina Health Centers. Based on the phenomenon of interest, recommendations from qualitative studies, and practical considerations such as data and resource needs, 14 key informants were purposively selected based on predefined criteria, such as training, experience, education, and position they held.

We included and reviewed the central triage register of all adults aged 30 years and older, screened for hypertension to identify patients with initially elevated BP. We included all actively followed patients with hypertension for BP control assessment. Also, we randomly took a convenient sample of 20 patients’ records to assess data completeness and the agreement between cohort register and surveillance reporting platforms. For sensitivity calculation, we compared the proportions of hypertensive cases captured by the surveillance system with those revealed by the survey findings. Finally, we took all individuals having initially raised BP and later confirmed to truly have hypertension to determine positive predictive value (PPV).

### Data Collection Procedures

We followed the Centers for Disease Control and Prevention’s updated guidelines for evaluating public health surveillance systems to assess the usefulness and 9 attributes of the surveillance system [21]. In addition, we adapted the standards for communicable disease surveillance and response systems evaluation to assess the core and supportive surveillance functions such as case detection, investigation and confirmation, communication and reporting, routine data analysis and interpretation, the availability of surveillance platforms, training, and supportive supervision and feedback system [22].

Data were collected using a semistructured questionnaire through face-to-face interviews, observations, and record reviews. Simplicity, flexibility, stability, acceptability, and usefulness were assessed via key informant interviews. A semistructured interview guide was developed in English and translated into the local language, Oromifa. The interview guide was designed in a way that allowed free responses as well as choices from predefined categories, for example, yes or no response-option questions. After the informant consent was obtained from each key informant, the lead author (ADG) conducted 14 face-to-face interviews. Interviews were audio-recorded, notes were taken, and each interview lasted 15 to 30 minutes.

We assessed data analysis and interpretation practice, availability of surveillance guidelines and standards, supervision and feedback, and timeliness via observations. Using medical record reviews, hypertension screening and case detection, BP control, investigation and confirmation, BMI screening, data quality, PPV, and representativeness were assessed. Both medical record reviews and observations were conducted by trained Bachelor of Science (BSc) degree nurses.

### Operational Definitions

#### Usefulness

Usefulness refers to the relevance of the surveillance system to detect diseases to injuries, estimate its magnitude, and assess the effects of prevention and control efforts [21]. The system was considered “useful” if it provided accurate and timely data on the magnitude of hypertension in the community, and supported decision-making for health interventions.

#### Simplicity

Simplicity refers to the ease of the surveillance structures and the ease of operation. A “simple” system was defined as one that required minimal resources, training, time to operate, and avoids complexity about in its information flow.

#### Acceptability

Acceptability refers to the willingness of surveillance personnel and organizations to participate in the surveillance system. The system was considered “acceptable” if the surveillance staff was willing to implement the system. This was assessed by the status of their active participation in case detection, adherence to case definition, completeness, and timeliness of reporting.

#### Timeliness

Timeliness reflects the speed between steps in a public health surveillance system. Timeliness was calculated by assessing how many of the expected reports have been submitted within the system-prescribed time frame. The system was considered “timely” if all expected reports have been submitted within a system-defined time frame.

#### Data Quality

Data quality refers to the completeness and validity of the recorded data in the public health surveillance system. It was assessed in terms of data completeness, expressed as the proportion of reports submitted to the next level of health authority, the completeness of surveillance platforms, measured by the proportion of surveillance forms fully recorded, and the validity of the data recorded.

#### Flexibility

Flexibility refers to the ability of the surveillance system to adapt to the changing information needs or operating conditions. A system was considered “flexible” if it could easily accommodate changing needs, such as addition of new data variables, modification of data collection process, or adjustment of reporting mechanisms.

#### Representativeness

Representativeness refers to the ability of surveillance system to describe the occurrence of health-related events by the person, place, and time characteristics. It was assessed by determining on whether the surveillance system provided an accurate description of hypertension in the target population. The system was considered “representative” if it included a balanced representation of the surveillance population.

#### Stability

Stability refers to the ability to collect, manage, and provide data properly without failure, and the ability to be operational when needed. The system was considered “stable” if it operated without significant downtime or data loss during its restructuring or changing needs.

#### Sensitivity

Sensitivity refers to the ability of the surveillance system to accurately identify disease or condition under surveillance. The system was considered “sensitive” if it could capture hypertensive cases that actually have a disease. This was assessed by comparing the proportion of patients with hypertension captured by the surveillance system and those identified by the baseline survey findings.

#### PPV

PPV refers to the proportion of reported cases that actually have the health-related event under surveillance [21]. PPV was assessed by calculating the proportion of surveillance-reported cases that were confirmed as true cases of hypertension. The system was considered “positively predictive” if could truly identify hypertensive cases.

### Data Quality Control

Before the actual data collection, we discussed with key informants to ensure that the evaluation was able to address important questions and gather credible evidence. During each visit of data collection, we briefed participants on the purpose and objective of the evaluation, underscoring that it is not an individual performance evaluation. The entire data collection process was closely monitored by research supervisors and principal investigators.

### Data Analysis

The quantitative data were coded, cleaned, and analyzed via SPSS software version 25.0 (IBM Corp) while the qualitative data were analyzed via the following methods. First, we transcribed the audio-recorded data and carefully read the text to obtain a general sense of the content. Second, we identified condensed meaning units and developed initial code books. Code books were designed in a way that they are flexible and devised when novel themes emerged. After the open coding, we created subcategories that merged codes with similar meanings, and subcategories were then combined to establish categories. The data were subsequently analyzed manually, narrated, and summarized via thematic analysis.

### Ethical Considerations

The study was approved by the Afar Public Health Institute (approval APH/25/21). We also obtained a collaboration letter from Ethiopia’s Ministry of Health, Oromia’s Public Health Institute, and Mojo City’s Administration Health Office. Before commencement of interview, informed consent was obtained from the participant to ensure their understanding and agreement to the recording process. After each participant understood the purpose, potential risks and benefits of the study, they were orally consented for participation in the study. The participation process was entirely voluntary and no monetary or material compensation was provided to the participants. Such consent and compensation processes were part of the study protocol approved by the ethical approval committee. The data and all collected information were delinked, deidentified, and anonymyzed for confidentiality. They were also kept in a secure file cabinet.

## Results

### Study Characteristics

The study invited 14 key informants for interviews, and all were willing to provide an interview. Of these key informants, 4 (29% held master’s degrees in public health, 4 (29%) were public health officers, and 6 (42%) were BSc degree nurses, of whom 4 (29%) were surveillance officers, and 2 (14%) were facility heads (Table 1).

**Table 1.** Key informant characteristics. Evaluation of a sentinel hypertension surveillance system in Mojo city, Eastern Shewa, Oromia, Ethiopia, 2022.

Key informant characteristics	Frequency, n (%)	
Title or degree		
Master of Public Health	4 (29)	
Public Health Officer	4 (29)	
Bachelors of Science degree nurses	6 (42)	
Location		
Mojo Health Center	5 (36)	
Haachaltu Gudina Health Center	5 (36)	
Mojo City Health Office	2 (14)	
Oromia Regional Health Bureau	2 (14)	
Work position		
Hypertension screening unit	2 (14)	
Hypertension focal persons	4 (29)	
Surveillance officers	4 (29)	
Data Management	2 (14)	
Facility heads	2 (14)	
Hypertension training		
Trained for hypertension	6 (43)	
Untrained for hypertension	8 (57)	
Work experience		
Junior level (<2 y)	2 (14)	
Intermediate level (2-<5 y)	4 (29)	
Senior level (≥5 y)	8 (57)	

### Description and Operation of the Surveillance System

The hypertension surveillance system in the Mojo City is a multitiered framework involving various health care providers and administrative personnel. Public health officers and BSc nurses are responsible for identifying and diagnosing hypertension, and initiating antihypertensive treatment. Health information technicians collect and report data, while the administrative staffs oversee data aggregation, analysis, and interpretation and use for action.

Based on the national clinical guidelines, surveillance system in Mojo City defines hypertension with a systolic blood pressure (SBP) of ≥140 mmHg or a diastolic blood pressure (DBP) of ≥90 mmHg in 2 consecutive visits. BP control is defined as achieving an SBP of <140 mmHg and a DBP of <90 mmHg in 2 consecutive visits, with the specific targets of <130/90 mmHg in patients having comorbidities such as diabetes, chronic kidney disease, and cardiovascular diseases.

**Figure 1.**
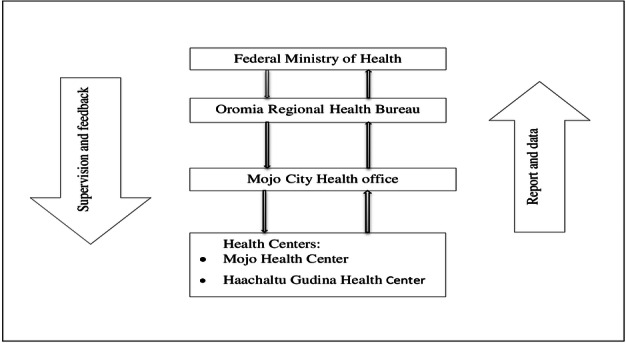
The information flow and reporting scheme for a sentinel hypertension surveillance system in Mojo city, Eastern Shewa, Oromia, Ethiopia, 2022.

The surveillance system uses both electronic and paper-based reporting platforms to facilitate efficient data flow between health care providers and surveillance officers. The information flow follows a bottom-up approach, with data transmitted from health centers to the city health office and then to the regional health bureau (Figure 1).

### Case Detection

Between September 1, 2021, and July 30, 2022, a total of 3760 adults aged 30 years and older were screened for hypertension. Among those screened, 553 (14.7%) individuals were presented with raised BP, and 512 (13.6%) individuals were confirmed to have hypertension. Of the patients confirmed with hypertension, 76 (14.8%) patients were lost, while 436 (85.2%) were receiving antihypertensive treatment. Only 214 (49%) patients who were on antihypertensive follow-up had achieved controlled BP.

### Investigation and Confirmation

A systematic approach was employed to confirm individuals with initially raised BP. Nurses and health officers oversee BP screening and BP confirmation. All patients with raised BP were reassessed for confirmation according to the national clinical guidelines. However, the initiation of antihypertensive therapy lacked essential investigations. Specifically, only 9.7% (50/512) of patients received a baseline renal panel, despite it being a crucial step outlined in the national clinical guidelines and patients may be at higher risk of undiagnosed kidney damage, which could lead to worse outcomes, including kidney failure and cardiovascular complications. The test is essential for evaluating kidney function, detecting early signs of kidney damage, assessing potential comorbidities related to hypertension, such as chronic kidney disease, tailoring treatment plans, and preventing the progression of hypertension-related renal complications. Furthermore, BMI screening, another essential component of hypertension management was not systematically conducted for all patients, with only 25.8% (132/512) of patients with hypertension undergoing this assessment.

### Communication and Reporting System

The surveillance system uses Health Management Information System (HMIS) framework of reporting diseases in Ethiopia. Health centers submit reports to Mojo city administration Health Office every 21st-26th of each month. Similarly, Mojo city’s administration Health Office subsequently compiled and sent to Oromia Regional Health Bureau within the same time frame. The Oromia Regional Health Bureau further transmits reports to the Federal Ministry of Health within the same reporting schedule. This type of surveillance system was fully implemented throughout the year and maintained through both electronic and paper-based reporting system (Figure 1).

### Data Analysis, Interpretation, Action, and Response

Health facilities did not conduct routine data analysis, resulting in hypertension data that lacked specific information regarding time, place, and person characteristics. In addition, there were no established action thresholds for the interpretation of data or for formulating appropriate responses.

### Availability of Surveillance Guidelines, Standards, and Other Resources

The surveillance system in the Mojo City ensured the continuous supply of essential surveillance forms, standards, and guidelines. Both health facilities had sufficient back up store of tally sheets, intake forms, follow-up forms, referral forms, and cohort registers. However, health facilities had the shortage of appointment forms and tablets for SIMPLE App (Resolve To Save Lives), a smart app designed for tracking patient follow-up.

### Supervision, Feedback, and Training

Neither Ethiopia’s Federal Ministry of Health nor the Oromia Regional Health Bureau provided written feedback regarding the performance of health facilities. Only 6 out of 14 (43%) of key informants received hypertension training. In addition, the heads of health facilities expressed concerns about inadequate training and backup staff to manage staff turnover and annual leave.

### Usefulness and Surveillance System Attributes

#### Usefulness

All 14 key informants (100%) indicated that the surveillance system was valuable, as its record-keeping capabilities documented data that highlighted the magnitude of hypertension in the area and helped them to integrate hypertension service at the community-level involving community health extension workers. One participant noted:

*I found the existing system immensely important because it helped us understand the extent of the hypertension problem in the area, evaluate our efforts in prevention and control, and improve chart keeping. I believe the lessons learned will be instrumental for further program expansion.* [Participant 5, Head of Health Care Site X]

#### Acceptability

The active participation of surveillance staff in case detection, adherence to case definitions, and report completeness and timeliness demonstrated their full commitment to implementing the surveillance system. One participant expressed:

*I am very happy being the hypertension focal person at the health facility and part of this sentinel surveillance system. I am fully committed to hypertension screening and case detection, utilizing case definitions, and providing timely and complete surveillance reports*. [Participant 6, Hypertension Focal Person from Facility Y]

#### Simplicity

All 14 (100%) of key informants agreed that the current case definition was clear, easy to understand, and apply, and that reporting platforms are user-friendly, requiring average completion time of 10‐15 minutes. They noted that the route of information flow was clear and well-delineated. They also reported that the SIMPLE App is intuitive and required minimal or no training for data entry and updates. One participant stated:

*... Without any reservation, in my view, the hypertension case definition is easy for me to understand and apply, and I am able to complete surveillance forms within a matter of 10 minutes. I am also clear with for whom to report, reporting period, and formats used for reporting.* [Participant 9, surveillance officer from health care site Y]

#### Flexibility

In total, 10 out of 14 key informants (71%) thought that the current reporting form could be easily adapted to new health events, and that case definition of hypertension was flexible enough to accommodate changes. The system has previously experienced significant changes in case definition and its treatment targets, and that they did not face much difficulty in both aspects. This adjustment shifted the focus from individuals aged 18 years and older to those aged 30 years and older for BP screening, as well as updated BP control treatment targets of BP below 130/90 mmHg for patients with hypertension having diabetes, chronic kidney disease, and cardiovascular conditions. One participant stated:

*I think we can modify the current case definition slightly to allow for other events. Moreover, I believe the current system can operate with varying funding sources, as it was once well established.* [Participant 3, Nurse at the Hypertension Screening Unit from Health Care Site X]

#### Stability

Out of 14, 7 participants (50%) of the surveillance staff noted that restructuring during the down times of the computer system breakage did not affect the operation of surveillance system and that surveillance system was able to continue to collect, manage, and provide data properly without failure. However, the other half-expressed concerns regarding the adequacy of trained backup staff and the program’s dependency on a partner, citing insufficient input from local and regional governments. One participant noted:

*There is a common practice in Ethiopia: programs supported by partners and external sources tend to be more effective, but the challenge arises when they are left to operate independently. Our system is similarly dependent on the partner; how could it continue if the partner withdraws support? I have serious concerns about its sustainability.* [Participant 2, Surveillance Officer from Administrative Institution B]

#### Timeliness

**Figure 2.**
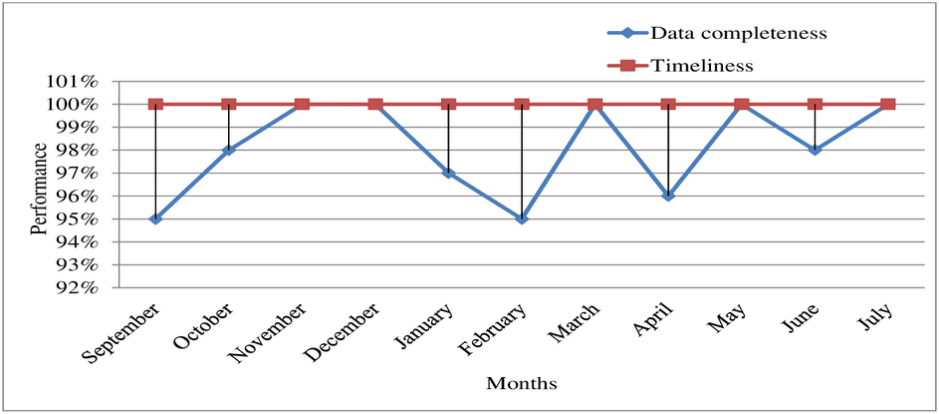
The report timeliness and completeness in a hypertension sentinel surveillance system in Mojo City, East Shewa Zone, Oromia region, Ethiopia, 2022.

For all 11 months included in the evaluation, reports between each reporting units in the surveillance chain, specifically from health facilities to the Mojo City Administration Health Office, from the Mojo City Administration Health Office to the Oromia Regional Health Bureau, and from the Oromia Regional Health Bureau to the Federal Ministry of Health were submitted according to HMIS framework for reporting diseases in Ethiopia between the 21st and 26th of each month (Figure 2).

#### Data Quality

Of the 20 patient medical records reviewed, 17 (85%) patient charts and follow-up forms were complete, neat, and filled in an easily understandable manner. The agreement between patient charts and cohort register was observed for all these patients. The completeness of the reports over the 11 months of review was 98% (Figure 2).

#### Representativeness

By screening 3760 adults aged 30 years and older from a corresponding target population of 17,000 inhabitants, the surveillance system reached 22% of the target population. The system identified 512 patients with hypertension, of whom 352 (69%) were reported from the Mojo Health Center, and 270 (52.9%) were females.

#### PPV and Sensitivity

The BP screening and confirmation practices were taken to calculate PPV. Of the 553 patients with initially raised BP levels, that is, SBP ≥140 mmHg or DBP ≥90 mmHg, 512 (92.6%) individuals truly had hypertension after reassessment (PPV). Similarly, to assess sensitivity, we used a 30% prevalence of hypertension from a baseline facility-based survey conducted in 2020 using a representative sample as a benchmark and compared the current proportion of patients with hypertension identified by the surveillance system (512/3760, 13.6%). Thus, the sensitivity of the surveillance system was 45.3% (Table 2).

**Table 2.** A performance comparison table showing system attributes in a hypertension surveillance system in Mojo City, East Shewa Zone, Oromia region, Ethiopia, 2022.

System attributes	Haachaltu Gudina health center	Mojo health center	Mojo city health office	Conclusion
Usefulness	Useful	Useful	Useful	Useful
Acceptability	Acceptable	Acceptable	Acceptable	Acceptable
Simplicity	Simple	Simple	Simple	Simple
Flexibility	Flexible	Flexible	Flexible	Flexible
Stability	Unstable	Unstable	Unstable	Unstable
Representativeness	(1650/7534, 21.9%)	(2110/9466, 22.3%)	(3760/17,000, 22%)	Unrepresentative
Data completeness	(505/520, 97.1%)	(552/559, 98.7%)	(1057/1079, 98%)	High
Timeliness	(11/11, 100%)	(11/11,100%)	(11/11,100%)	High
Sensitivity	43.5%	47.1%	45.3%	Low
Positive predictive value	91.8%	93.4%	92.6%	High

## Discussion

### Principal Findings

We followed the Centers for Disease Control and Prevention’s updated guidelines for evaluating public health surveillance systems to assess on whether the existing surveillance system in Mojo City operated effectively and efficiently. The usefulness and 9 system attributes were streamlined in this evaluation. Accordingly, the surveillance system in Mojo city was useful, simple to operate, flexible to changing needs, acceptable by surveillance staff, has higher PPV but less sensitive, unrepresentative, and unstable.

While the surveillance system in Mojo City was fully operational throughout the year, it lacked regular supportive supervision and feedback mechanisms. There is need to institutionalize periodic evaluations, enhance the system’s functionality, and foster a continuous improvement. Public health surveillance system should be periodically evaluated to provide scientifically sound and epidemiologically interpreted feedbacks for performance and further for its improvement [22,23].

All surveillance staff expressed willingness to implement the system and noted that it was both useful and simple. Half perceived the surveillance system stable, while 71% (10/14) thought that it was flexible. Similarly, the performance of public health surveillance system in Dangila, Ethiopia, was found flexible and useful [24]. Furthermore, a hypertension surveillance system evaluation conducted in Indonesia at 2 health centers and 1 administrative office reported an overall low stability, simplicity, acceptability, and data quality, but the researchers were unable to assess flexibility [25]. However, unlike this study, researchers relied on data collection, storage, and reporting to assess stability, rather than using key informant interviews [25].

Given that 3760 adults aged ≥30 years were screened for hypertension out of a corresponding population of 17,000 inhabitants, the surveillance system in a Mojo City demonstrated low representativeness, having reached only 22% of the population. However, in a hypertension surveillance evaluation conducted in Indonesia, they were unable to assess representativeness of surveillance system [25]. The low representativeness in the present surveillance system may be attributed to low community awareness and routine BP screening, and regular medical checkup tradition, which could leave significant numbers of undiagnosed patients in the community. In this system, health centers collect data and report it to the Mojo City Health Office and the Oromia Regional Health Bureau. However, health facilities did not conduct routine data analysis or interpretation, nor did they use the information to inform action. Data collection necessitates analysis because the goal of surveillance is to use the information for action, reduce morbidity and mortality, and to improve overall health [22,23]. If the collected data are not followed by regular analysis and the application of information for action, it results in the mere consumption of resources [26].

Public health surveillance systems should operate in a manner that facilitates effective communication and dissemination of health and health-related data, enabling decision-makers at all levels to readily understand the implications of the information. Timeliness of reporting is a crucial factor for the effective communication and dissemination of health and health-related data. The report timeliness in the current surveillance system was 100%, which aligns with the Ethiopian national target of 95% [27] and the WHO target of 80% [28]. In addition, data completeness was 98%, consistent with the national target of over 95% for report completeness [27] and the WHO target of 80% [28].

The sensitivity and PPV of a surveillance system depend on the design of the case definition and its clarity among stakeholders [29]. The surveillance system in Mojo City exhibited a sensitivity of 45.3%, capturing only 45.3% of hypertension cases and more than half (54.7%) of the individuals with hypertension may be missed by the system. It is less sensitive than the hypertension surveillance system in Bogor City, Indonesia, which has a sensitivity of over 80% [30]. The low sensitivity may be attributed to low health care service utilization and suboptimal BP screening practice, as only 22% of the surveillance population was screened for BP. In a hypertension surveillance system evaluation at the Jombang District Health Office, Indonesia, they were unable to calculate sensitivity due to the lack of baseline survey data for comparison. However, the PPV of our surveillance system was 92.6%, indicating that the system correctly identifies 92.6% of true cases of hypertension. It is more predictive when compared with other hypertension surveillance systems in Dangila, Ethiopia which has PPV of 11% [24] and Jombang District Health Office of the other city in Indonesia which has PPV of 59.4% [25].

### Limitations of the Study

The limitations of this evaluation are as follows. First, the results may have been subject to potential social desirability bias, as surveillance stakeholders may have provided favorable responses especially qualitative attributes such as acceptability and simplicity. The surveillance stakeholders may have been inclined to provide more favorable responses to questions about these attributes, potentially overestimating the true level of satisfaction or ease with the surveillance system. To mitigate this, we briefed them on the purpose and objectives of the evaluation at each visit before commencing data collection. Second, reliance on a convenience sample of 20 patients may not have provided a representative assessment of data validity. Third, although this evaluation followed the Centers for Disease Control and Prevention’s updated guidelines for evaluating public health surveillance systems as applied in previous studies conducted in different settings to gather credible evidence on the performance of the surveillance system, it is primarily relied on responses from key informant interviews rather than objective quantitative data collection, storage, and reporting, unlike other studies. Finally, the availability of the limited data in the field led to insufficient understanding of surveillance systems in similar contexts and a few of interpretations were made compared with surveillance systems in other country which may have structural and contextual difference with our surveillance system.

### Conclusion

The sentinel hypertension surveillance system in Mojo demonstrated effective implementation throughout the year. The surveillance staff felt that it was helpful in determining the magnitude of hypertension in the area as well as for improving documentation and patient record keeping practices. It was found to be simple, flexible, acceptable, and have a higher PPV. The data quality and reporting timeliness were consistent with national and WHO surveillance standards. However, it was less sensitive, less representative, and unstable.

The findings underscore the need for institutionalization of periodic evaluations and supportive supervision mechanisms to assess the system’s performance, provide feedback, and identify areas for improvement. The system should implement routine data analysis and interpretation mechanisms based on epidemiological principles to interpret the collected data, identify trends, and target tailored public health interventions. Continuous staff training on data collection, surveillance protocols, and the operation of the surveillance system should be designed to enhance stability. Furthermore, designing of government-driven sustainability strategies, such as establishment of dedicated public health financing system and consistent resource allocation and capacity building, and cross-sectoral collaborations with local governments, could reduce reliance on external donors and enhance the sustainability of the surveillance system. Efforts should also focus on community awareness creation activities to improve sensitivity and representativeness of the surveillance system. Finally, if resources permit, scaling the system into a nationwide program could significantly enhance hypertension surveillance across Ethiopia.

## Abbreviations

BP: blood pressure
BSc: Bachelors of Science
DBP: diastolic blood pressure
HMIS: Health Management Information System
NCD: noncommunicable disease
PPV: positive predictive value
SBP: systolic blood pressure
WHO: World Health Organization

## References

[1] Mensah GA, Roth GA, Fuster V (2019). The global burden of cardiovascular diseases and risk factors: 2020 and beyond. J Am Coll Cardiol.

[2] Mills KT, Stefanescu A, He J (2020). The global epidemiology of hypertension. Nat Rev Nephrol.

[3] Hypertension key fact sheet. World Health Organization.

[4] Kearney PM, Whelton M, Reynolds K, Muntner P, Whelton PK, He J (2005). Global burden of hypertension: analysis of worldwide data. The Lancet.

[5] Zhou B, Carrillo-Larco RM, Danaei G (2021). Worldwide trends in hypertension prevalence and progress in treatment and control from 1990 to 2019: a pooled analysis of 1201 population-representative studies with 104 million participants. The Lancet.

[6] Molla M (2015). Systematic reviews of prevalence and associated factors of hypertension in Ethiopia: finding the evidence. SJPH.

[7] Shiferaw F, Letebo M, Misganaw A, Feleke Y, Gelibo T, Getachew T (2018). NCDs in Ethiopia: Disease burden, gaps in health care delivery and strategic directions. Ethiopian Journal of Health Development.

[8] Misganaw A, Mariam DH, Ali A, Araya T (2014). Epidemiology of major non-communicable diseases in Ethiopia: a systematic review. J Health Popul Nutr.

[9] Jung M, Jembere GB, Park YS (2021). The triple burden of communicable and non-communicable diseases and injuries on sex differences in life expectancy in Ethiopia. Int J Equity Health.

[10] Federal Democratic Republic of Ethiopia, Ministry of Health National strategic action plan (NSAP) for prevention and control of noncommunicable diseases in ethiopia, 2014-2016. https://www.iccp-portal.org/system/files/plans/ETH_B3_National%20Strategic%20Action%20Plan%20%28NSAP%29%20for%20Prevention%20and%20Control%20of%20Non-Communicable%20Diseases%20-%20Final.pdf.

[11] World Health Organization Non-Communicable Diseases Country Profiles 2018.

[12] Legese N, Tadiwos Y (2020). Epidemiology of hypertension in Ethiopia: a systematic review. Integr Blood Press Control.

[13] Kebede B, Chelkeba L, Dessie B (2021). Rate of blood pressure control and its determinants among adult hypertensive patients at Jimma University Medical Center, Ethiopia: Prospective cohort study. SAGE Open Med.

[14] Horsa BA, Tadesse Y, Engidawork E (2019). Assessment of hypertension control and factors associated with the control among hypertensive patients attending at Zewditu Memorial Hospital: a cross sectional study. BMC Res Notes.

[15] Gebremichael GB, Berhe KK, Zemichael TM (2019). Uncontrolled hypertension and associated factors among adult hypertensive patients in Ayder comprehensive specialized hospital, Tigray, Ethiopia, 2018. BMC Cardiovasc Disord.

[16] Animut Y, Assefa AT, Lemma DG (2018). Blood pressure control status and associated factors among adult hypertensive patients on outpatient follow-up at University of Gondar Referral Hospital, northwest Ethiopia: a retrospective follow-up study. Integr Blood Press Control.

[17] WHO Noncommunicable Disease Surveillance, Monitoring and Reporting Surveillance of noncommunicable diseases.

[18] Ethiopian Health and Nutrition Research Institute (2012). Public Health Emergency Management Centre Public Health Emergency Management guidelines for Ethiopia, 2012.

[19] Ethiopian Public Health Institute Public Health Emergency Management Center, Federal Democratic Republic of Ethiopia (2022). Public Health Emergency Management Guideline for Ethiopia.

[20] WHO regional office for Africa (2019). Ethiopia sets to improve hypertension prevention and control at primary health care level.

[21] Centers for Disease Control and Prevention (CDC) (2001). Updated guidelines for evaluating public health surveillance systems recommendations from the guidelines working group. https://www.cdc.gov/mmwr/preview/mmwrhtml/rr5013a1.htm.

[22] WHO (2006). Communicable disease surveillance and response systems. guide to monitoring and evaluating. https://iris.who.int/bitstream/handle/10665/69331/WHO_CDS_EPR_LYO_2006_2_eng.pdf.

[23] LANGMUIR AD (1963). The surveillance of communicable diseases of national importance. N Engl J Med.

[24] Alemu T, Gutema H, Legesse S, Nigussie T, Yenew Y, Gashe K (2019). Evaluation of public health surveillance system performance in Dangila district, Northwest Ethiopia: a concurrent embedded mixed quantitative/qualitative facility-based cross-sectional study. BMC Public Health.

[25] Lestari D (2021). Evaluation of monitoring hypertension case based on nine surveillance attributes in Jombang District Health Office in 2018. ijph.

[26] Foege WH, Hogan RC, Newton LH (1976). Surveillance projects for selected diseases. Int J Epidemiol.

[27] The Federal Democratic Republic of Ethiopia, Ministry of Health Health Sector Transformation Plan Health Sector Transformation Plan 2015/16 - 2019/20 (2008-2012 EFY) August 2015. Food and Agriculture Organization of of the United Nation.

[28] World Health Organization (2019). Integrated disease surveillance and response technical guidelines: booklet three: sections 4, 5, 6, and 7. https://apps.who.int/iris/bitstream/handle/10665/312362/WHO-AF-WHE-CPI-03.2019-eng.pdf.

[29] PennState Eberly College of science STAT 507 | Epidemiological Research Methods. STAT ONLINE | Department of Statistics.

[30] Amalia D, Kusnadi B, Bantas K The evaluation of hypertension surveillance system based on primary health care in bogor city. Indonesia: The 8th National Scientific Conference on Epidemiology.

